# 

**Published:** 1891-09

**Authors:** 


### Society Meetings.

---

The American Public Health Association will hold its nineteenth annual meeting at Kansas City, Tuesday, Wednesday, Thursday, and Friday, October 20, 21, 22, and 23, 1891.

The following topics have been chosen for consideration at this meeting: (1) Sanitary Construction in House Architecture; (2) Railroad Sanitation; (3) Meat Supplies; (4) Milk Supplies of Cities; (5) Arsenical Papers and Fabrics; (6) Isolation Hospitals for Infectious Diseases in Cities.

Among its officers and members of committees, we notice the names of several Buffalo physicians: Dr. Edward Clark is a member of the executive committee; also on the special committee on the Disposal of Garbage and Refuse. Dr. F. P. Vandenbergh is one of the committee on the Pollution of Water Supplies. Dr. W. D. Greene is on the committee on the Cause and Prevention of Diphtheria.

THE MISSISSIPPI VALLEY MEDICAL ASSOCIATION will hold its seventeenth annual session at the Pickwick Theatre, Washington and Jefferson avenues, St. Louis, October 14th, 15th, and 16th. A full programme of interesting papers has been prepared and provision has been made for the fullest, freest and most complete discussion of the same. Representative men from various sections of the country have been invited to open the discussions. The local profession of St. Louis is a unit to the end that every visiting physician shall be received and welcomed in a regular warm-hearted St. Louis style. The same qualifications for membership are requisite in this Association as for the American Medical Association, the former being subordinate to the latter. All eligible physicians and their friends, together with their wives and families, are most cordially invited to visit St. Louis and enter into the scientific work and the social pleasures as they may desire.

I. N. LOVE, M. D., Chairman,
*Committee Arrangements.*

---

THE THIRD ANNUAL MEETING OF THE TRI-STATE MEDICAL ASSOCIATION will convene in Turner Hall, Chattanooga, Tenn., Tuesday, October 27, 1891, and continue in session three days. Indications are that it will be one of the largest medical meetings ever held in the South. Representative physicians from all sections will be present.

All who desire to read papers should send title to the Secretary of the Association, Dr. Frank Trester Smith, before September 1st. In due time a circular will be issued, giving a complete list of all papers and names of exhibitors who apply for space before October 1st.

W. L. GAHAGAN,
*Sec'y of Executive Com.,*
P. O. Box 542. Chattanooga, Tenn.

---

THE AMERICAN ASSOCIATION OF OBSTETRICIANS AND GYNECOLOGISTS will hold its fourth annual meeting, at the New York Academy of Medicine, 17 West Forty-third street, in the City of New York, Thursday, Friday, and Saturday, September 17, 18, and 19, 1891, under the presidency of Dr. Adam H. Wright, of Toronto. All physicians interested in the discussion of subjects pertaining to Abdominal Surgery, Obstetrics, and Gynecology, are invited to attend without further formal notice. By order of the Executive Council.

The Canadian Medical Association will hold its twenty-fourth annual meeting in Montreal, on Wednesday, Thursday, and Friday, 16th, 17th, and 18th September. Members desirous of reading papers or presenting cases will kindly communicate with the secretary, Dr. H. S. Birkett, 123 Stanley street, Montreal, as to title of paper or nature of case, as early as possible. Arrangements are being made with the various railway and steamboat companies, whereby members can obtain return tickets at considerably reduced rates.

---

The American Orthopedic Association will hold its fifth annual meeting in the new reception room of the Arlington Hotel, Washington, D. C., Tuesday, Wednesday, Thursday, and Friday, September 22, 23, 24, and 25, 1891, under the presidency of Dr. A. B. Judson, of New York. An interesting programme, consisting of sixty-seven papers on important subjects by able men throughout this and foreign countries, has been prepared, and a profitable meeting may be predicted.

---

The American Electro-Therapeutic Association will hold its first annual meeting at the Hall of the College of Physicians, corner Locust and Thirteenth streets, Philadelphia, Pa., Thursday, Friday, and Saturday, September 24, 25, and 26, 1891, under the presidency of Dr. G. Betton Massey.

Physicians interested in the discussion of electricity in medicine are invited to attend without further notice.

### BOOKS AND PAMPHLETS RECEIVED.

A Text-Book of Practical Therapeutics. With especial reference to the Application of Remedial Measures to Disease and their Employment upon a Rational Basis. By Hobart Amory Hare, M. D., B. Sc., Professor of Therapeutics and Materia Medica in the Jefferson Medical College of Philadelphia; Physician to St. Agnes' Hospital and the Medical Dispensary of the Children's Hospital; Laureate of the Royal Academy of Medicine in Belgium; of the Medical Society of London; Secretary of the Convention for the Revision of the United States Pharmacopeia of 1890. Second edition. Enlarged and thoroughly revised. Cloth and leather, 8vo, pp. xii.—658. Philadelphia: Lea Brothers & Co. 1891.

Practical Pathology and Morbid Histology. By Heneage Gibbes, M. D., Professor of Pathology in the University of Michigan; formerly Lecturer on Normal and Morbid Histology in the Medical School of the Westminster Hospital, London; formerly Curator of the Anatomical Museum, King's College, London. Illustrated with sixty photographic reproductions. Octavo, pp. xii.—320. Philadelphia: Lea Brothers & Company. 1891.

Transactions of the Medical Society of the State of New York for the year 1891. Edited by Frederic C. Curtis, M. D., Secretary of the Society. 8vo, pp. viii.—560. Published by the Society. 1891.

Index-Catalogue of the Library of the Surgeon-General's Office, United States Army. Authors and Subjects. Vol. XII. Reger-Shuttleworth. Quarto, pp. 1004. Compiled by John S. Billings, Surgeon United States Army. Washington: Government Printing Office. 1891.

Report on Cholera in Europe and India. By Edward O. Shakespeare, A. M., M. D., Ph. D., of Philadelphia, United States Commissioner. Transmitted to the Secretary of State. Quarto, pp. xxvi.—945.

Stories of a Country Doctor. By Willis P. King, M. D., member of the American Medical Association; member and ex-President of the Missouri State Medical Association; Assistant Chief Surgeon of the Missouri Pacific Railway Company; formerly Lecturer on Diseases of Women in the Medical Department of the Missouri State University, and Professor of Diseases of Women in the Medical Department University of Kansas City; Lecturer on Orthopedic Surgery and Clinical Surgery in the University Medical College of Kansas City, etc., etc. Second edition. With Illustrations by T. A. Fitzgerald. Philadelphia: A. L. Hummel, M. D., publisher. 1891. Price, $1.00, bound in cloth.

Transactions of the Texas State Medical Association. Twenty-third Annual Session held at Waco, April 28, 29, 30, and May 1, 1891. Edited by Hamilton A. West, M. D., Secretary. Galveston: Clarke & Courts. 1891.

Transactions of the Twenty-fourth Annual Session of the Medical Association of the State of Missouri held at Excelsior Springs, May 19, 1891. Edited by Lyman A. Berger, M. D., Secretary. Kansas City: Tiernan-Havens Printing Co. 1891.

Addresses, Papers, and Discussions in the Section of the Practice of Medicine and Physiology at the Forty-second Annual Meeting of the American Medical Association, at Washington, D. C., May 5, 6, 7, and 8, 1891. Chicago: Printed at the Office of the Association. 1891.

Practical Intestinal Surgery. Vol. II. By Fred. B. Robinson, B. S., M. D., Professor of Anatomy and Clinical Surgery, Toledo (Ohio) Medical College; *Physicians' Leisure Library*. Issued monthly. Detroit: George S. Davis. 1891. Price, cloth, 50 cents; paper, 25 cents.

The Mother's Hand-Book. A Practical Treatise on the Management of Children in Health and Disease. With an Appendix containing Articles on Diseases and Accidents that may suddenly happen to grown persons. By Levin J. Woollen, M. D. Richmond, Va.: Everett Waddy Co. 1891.

The Steps of the Cesarean Section. The Do's and Don't's. By H. A. Kelly, M. D. Reprint: American Journal of Obstetrics. Vol. XXIV. No. 9. 1891.

Proceedings and Addresses at a Sanitary Convention held at Charlevoix, Mich., August 14 and 15, 1890. Supplement and Reprint. Report Michigan State Board of Health. Lansing. 1891.

Third Annual Report of the Health Department, Mansfield, Ohio. 1890-91. By R. Harvey Reed, M. D., Health Officer, Mansfield. 1890.

Amputations in the Light of Prothetical Science. By Charles Truax, Chicago, Ill. Reprint.

Eye Diseases of the Unborn. By Julian J. Chisolm, M. D. Reprint: *Maryland Medical Journal*, May 30, 1891.

Can the Gynecologist Aid the Alienist in Institutions for the Insane? By I. S. Stone, M. D., Washington, D. C. Fellow American Association of Obstetricians and Gynecologists; Gynecologist to the Columbia Hospital, etc. Reprint: *Journal American Medical Association*, June 20, 1891.

The Direct Treatment of Diseased Tubes and Ovaries. By A. V. L.

Brokaw, M. D., St. Louis, Mo. Reprint: The *Medical Mirror*, July, 1891.

A New Intestinal Clamp. By A. V. L. Brokaw, M. D., Demonstrator of Anatomy and Operative Surgery, Missouri Medical College, etc. Reprint: *State Medical and Surgical Journal*, October, 1890.

Status Epilepticus. By G. R. Trowbridge, A. M., M. D., and C. B. Mayberry, A. M., M. D., Fellows of the American Academy of Medicine; Assistant Physicians State Hospital for the Insane, Danville, Pa. Reprint: *Journal of Nervous and Mental Diseases*. July, 1891.

Eleventh Annual Report of the State Board of Health of New York. Transmitted to the Legislature, February 20, 1891. Vol. I. and II. Albany: James B. Lyon. 1891.

A Compend of Human Physiology, especially adapted for the use of Medical Students. Sixth edition. By Albert P. Brubaker, M. D., Professor Physiology, Pennsylvania College of Dental Surgery; Demonstrator of Physiology in Jefferson Medical College, Philadelphia. Revised, enlarged, with new illustrations. Quiz Compends No. 4.

Injury to the Thoracic Duct with an Unique and Inevitable Death by Inanition. By Alvin Eyer, M. D., Cleveland, Ohio. Reprint: The *Medical Record*, August 1, 1891.

Vacation Time, with Hints on Summer Living. By H. S. Drayton, M. D. New York: Fowler & Wells Co., Publishers. 1891.

Extirpation of the Kidney for an Enormous Myxo-Sarcoma in a Child aged Three Years and Eight Months. By A. V. L. Brokaw, M. D., Demonstrator of Anatomy and Operative Surgery in the Missouri Medical College. Reprint: The *Medical News*, March 21, 1891.

Some Practical Points in the Technique of Abdominal and Pelvic Surgery. Same author. Reprint: St. Louis *Courier of Medicine*, March, 1891.

Resorcine as an Antipyretic. By W. Carroll Chapman, M. D., Louisville, Ky. Reprint.

Illustrative Cases of Congenital Club-Foot. By H. Augustus Wilson, M. D., Professor of Orthopedic Surgery in the Philadelphia Polyclinic and College for Graduates. Reprint: *Annals of Gynecology and Pediatrics*, June, 1891.

The Remarkable Effects of Diuretin in Removing Dropsy. By Robert H. Babcock, A. M., M. D., Professor in the Post-Graduate Medical School of Chicago. Reprint: The *New York Medical Journal*, July 11, 1891.

Comparative Data in the Treatment of Uterine Tumors. By Marie B. Werner, M. D., Philadelphia. Reprint: *Annals of Gynecology and Pediatrics*, July, 1891.

Adenoma Uteri. By Henry C. Coe, M. D., Gynecologist to the New York Cancer Hospital. Reprint: *American Journal of the Medical Sciences*, August, 1891.

The "Motive and Method" of Electricity in Pelvic Inflammation. By George F. Hurlbert, M. D., St. Louis, Mo. Reprint: The *Weekly Medical Review*, June 6, 1891.

Amputation (Bloodless) at the Hip Joint.—How Should it be Performed ? By Emory Lanphear, M. D., Surgeon to East Side Dispensary ; Professor of Surgery in the University Medical College, Kansas City, Mo. Reprint: *University Medical Magazine.*

Treatment of Penetrating Wounds of the Abdomen. By the same author. Reprint.

Trichina Spiralis. By H. M. Whelpley, M. D., Ph. G., F. R. M. S., Professor of Physiology and Histology, and Director of the Histological Laboratory of the Missouri Medical College, etc., etc.

The Status of the Hydrochlorate of Cocaine in Minor Surgery, as based upon the Experiments of Philadelphia Physicians. By Lewis H. Adler, Jr., M. D., Instructor in Rectal Diseases at the Philadelphia Polyclinic. Reprint: The *Therapeutic Gazette*, August 15, 1891.

Diphtheritic Paralysis. By Lewis H. Adler, Jr., M. D. Reprint: The *American Lancet*, October, 1889.

Normal Liquid Ergot in Enuresis Nocturna. By Lewis H. Adler, Jr., M. D. Reprint: The *Medical Age*, February 10, 1890.

Cocaine as a Local Anesthetic in Cases Requiring Amputation of the Digits. With report of fifty such operations in which the drug was employed. By Lewis H. Adler, Jr., M. D. Reprint: The *Medical Age*, July 25, 1891.

Laparo-Hysterorrhaphy as a Means of Cure of Cases of Extreme Prolapse, or Retro-Displacements of the Uterus. By W. J. Asdale, M. D., Pittsburgh, Pa. Reprint: *Journal American Medical Association*, July 11, 1891.

Arbeit aus der Kgl. Frauenklinik in Dresden. Über die Entbehrlichkeit der Scheiden-Ausspülungen und -Auswaschungen bei regelmässigen Geburten und über die grösstmögliche Verwerthung der äusseren Untersuchung in der Geburtshülfe. Fünfter Beitrag zur Verhütung des Kindbettfiebers mit einem Rückblick auf das Jahr 1890. Von Prof. Leopold und Dr. Goldberg. Sonderabdruck aus dem Archiv für Gynäkologie, Bund XL., Heft 3.

Zur Therapie hartnäckiger Retroflexion der Gebärmutter von B. S. Schultz, M. D. Nr. 24. (Vierundzwanzigstes Heft der ersten Serie.) Subskriptionspreis für eine Serie von 30 Vorträgen 15 Mark. Preis jedes einzelnen Heftes 75 Pf. Ausgegeben Mai 1891.

Post-Graduate Course of Lectures in the University of Toronto, delivered December 17, 18, and 19, 1890. By J. William White, M. D., Victor C. Vaughan, M. D., J. E. Graham, M. D., Robert Abbe, M. D., Alexander McPhedran, M. D., William Oldright, M. D., A. Primrose, M. B., B. E. McKenzie, M. D., and George A. Peters, M. D. Reprint: The *Canadian Practitioner*.

A Page of Medical History: Moliére and the Doctors. The President's Address. By W. J. Conklin, M. A., M. D., Dayton, Ohio. Reprint: Transactions of the Ohio State Medical Society. 1891.

The Insanity of Pubescence. By G. R. Trowbridge, A. M., M. D., Danville, Pa. Fellow of the American Academy of Medicine ; Third Assistant Physician State Hospital for the Insane. Reprint: *The Alienist and Neurologist.* July, 1891.

HEALTH BULLETINS.

Newport, R. I., *Health Bulletin.* Reports of Deaths and Contagious Diseases. June and July, 1891.

New York State Board of Health. June, 1891.

Michigan State Board of Health. June and July, 1891.

Tennessee State Board of Health. July 20, 1891.

Abstracts of Sanitary Reports, U. S. Marine Hospital Service, Vol. VI. Nos. 16 to 34.

Notice to Contributors.—We are glad to receive contributions from every one who knows anything of interest to the profession. Articles designed for publication in the Journal should be handed in before the first day of the month. The Editors are not responsible for the views or opinions of contributors. All communications should be addressed to the Managing Editor, 284 Franklin Street, Buffalo, N. Y.

